# Galiximab (anti-CD80)-induced growth inhibition and prolongation of survival *in vivo* of B-NHL tumor xenografts and potentiation by the combination with fludarabine

**DOI:** 10.3892/ijo.2013.1986

**Published:** 2013-06-13

**Authors:** KANDASAMY HARIHARAN, PETER CHU, TRACEY MURPHY, DANA CLANTON, LISA BERQUIST, ARTURO MOLINA, STEFFAN N. HO, MARIO I. VEGA, BENJAMIN BONAVIDA

**Affiliations:** 1 Cancer Therapeutics, Biogen Idec, San Diego, CA 92122;; 2 Hematology/Oncology, Biogen Idec, San Diego, CA 92122;; 3 Department of Microbiology, Immunology and Molecular Genetics, David Geffen School of Medicine, Jonsson Comprehensive Cancer Center, University of California Los Angeles, Los Angeles, CA 90095, USA;; 4 Siglo XXI National Medical Center IMSS, Oncology Hospital, Oncology Research Unit, Mexico City 06720, Mexico

**Keywords:** galiximab, doxorubicin, fludarabine, non-Hodgkin’s lymphoma, tumor xenograft

## Abstract

Galiximab is a primatized monoclonal antibody that targets CD80 expressed on malignant B cells and is being studied in the clinic as a potential treatment for follicular NHL. We have recently reported that galiximab signals B-NHL cells *in vitro* and inhibits cell growth and sensitizes resistant tumor cells to apoptosis by chemotherapeutic drugs. This study was designed to validate the *in vitro* findings in *in vivo* in mice. Thus, we examined *in vivo* the antitumor activity of galiximab used alone and in combination with chemotherapeutic agents in SCID mice bearing human lymphoma xenografts. The *in vivo* antitumor effects of galiximab used alone and in combination with fludarabine or doxorubicin were determined in solid and disseminated human B-lymphoma tumors grown in SCID mice. Galiximab monotherapy *in vivo* demonstrated significant antitumor activity in a Raji lymphoma solid tumor model and in an SKW disseminated lymphoma tumor model. There was significant inhibition in tumor growth and prolongation of survival. *In vitro*, galiximab sensitized Raji cells to apoptosis by both fludarabine and doxorubicin. Tumor growth inhibition was significantly enhanced when the mice were treated with the combination of galiximab and fludarabine. These findings support the potential clinical application of galiximab in combination with chemotherapeutic drugs for the treatment of CD80-expressing hematological malignancies.

## Introduction

The non-Hodgkin’s lymphomas (NHLs) are a group of lymphoproliferative malignancies with divergent clinical courses. These disorders, which may originate from transformed B or T cells, can be grouped into aggressive and indolent categories (1). The aggressive NHLs have a relatively short natural history if untreated, but a proportion of patients can be cured with intensive chemotherapy (2). In contrast, the indolent lymphomas have a relatively long natural history, with median survival times as long as 10 years (3). During the early course of disease, the indolent lymphomas are generally responsive to radiation therapy, chemotherapy and monoclonal antibody (mAb)-based therapy and can be re-treated upon relapse. As the disease progresses, it can become refractory to therapy, with subsequent disease-free intervals becoming shorter (4). Although the NHLs have historically been treated with radiation therapy and/or chemotherapy, the standard of care has evolved to incorporate the use of rituximab, a mAb, which is directed against the CD20 antigen expressed on the surface of transformed B-lymphocytes (5).

CD80 (B7-1) is a cell-surface receptor of the immunoglobulin superfamily that is best known for its role in the costimulation of T-cell function causing T-cell proliferation, cytokine production and generation of cytotoxic T lymphocytes (6,7). The ligands for CD80 are CD28 and cytotoxic T-lymphocyte antigen 4 (CTLA-4, CD152) (8). CD80 is transiently expressed on antigen-presenting cells, T cells and activated B cells, but is constitutively expressed on the surface of many B-cell lymphomas (9–12). When cell-surface CD80 is cross-linked with anti-CD80 antibodies, cell proliferation is inhibited, pro-apoptotic molecules are upregulated and antibody-dependent cell cytotoxicity (ADCC) is induced (13). These studies provide the rationale for using an anti-CD80 mAb to treat lymphoma.

Galiximab is a primatized anti-CD80 mAb that has been tested as monotherapy in phase I/II clinical trials involving patients with relapsed/refractory follicular lymphoma (FL), producing an overall response rate of 11% and tumor reductions in 46% of patients (14). In a recent phase I/II clinical trial involving patients with relapsed or refractory FL, combined therapy with galiximab and rituximab yielded an overall response rate of 66% and a median progression-free survival of 12.1 months at the recommended phase II dose of galiximab (500 mg/m^2^) (15). A phase II CALGB trial of galiximab and rituximab was undertaken in patients with previously untreated FL. The regimen was well tolerated and efficacious in patients with low risk FLIPI scores (16).

Recently, we have reported that treatment of CD80^+^ B-NHL cells with galiximab resulted in cell signaling-induced inhibition of the constitutively activated NF-κB pathway. Inhibition of this pathway resulted downstream in the inhibition of both the metastasis-inducer and resistant transcription factor, SNAIL and the transcription and resistant factor Ying Yang 1 (YY1) and these inhibitory effects led to sensitization of tumor cells to apoptosis by drugs (e.g. CCDP and TRAIL) (17).

The present study sought to examine the *in vivo* efficacy of galiximab treatment on the growth of lymphoma tumor xenografts. In addition, since galiximab sensitizes tumor cells *in vitro* to apoptosis by drugs (17), we have also examined *in vivo* the antitumor effect of galiximab used in combination with fludarabine or doxorubicin. We show here that galiximab exhibits antitumor activity as a single agent in solid and disseminated human lymphoma xenografts in SCID mice. Further, the antitumor activity of galiximab was enhanced when used in combination with fludarabine.

## Materials and methods

### Cell lines

The human Epstein-Barr virus-transformed B-lymphocyte cell line, SKW6.4 (TIB-215) and the Burkitt lymphoma cell line, Raji (CCL-86), were obtained from ATCC (Manassas, VA, USA). Cells were cultured in RPMI-1640 medium (ATCC, 30-2001) supplemented with 10% fetal bovine serum (FBS; SH30071.03; HyClone, Logan, UT), L-glutamine 2 mmol/l, sodium pyruvate 1 mmol/l, and 1% penicillin-streptomycin at 37°C in an atmosphere of 5% CO_2_. All cells used in this study were within 15 passages after resuscitation. The cells were checked routinely by morphology and tested for mycoplasma contamination with the CELLshipper Mycoplasma Detection kit (Bionique Testing Laboratories).

### Animals

Female CB17 mice at 6–8 weeks of age with severe combined immunodeficiency (SCID) were used for *in vivo* tumor modeling studies (Charles River Laboratories Inc., Holister, CA) and were housed in polycarbonate cages using a HEPA-filtered, ventilated rack system (Allentown Inc., Allentown, NJ). All animal studies and procedures were performed under an institutionally approved protocol for animal care and use (IACUC #SD12-04; Biogen Idec, Cambridge, MA). The Biogen Idec animal facility is accredited by the Association for Assessment and Accreditation of Laboratory Animal Care International.

### Drugs/antibodies

Galiximab (IDEC-114) is a high-affinity, PRIMATIZED^®^, anti-CD80 immunoglobulin (Ig) G1, λ mAb. This antibody was obtained by immunizing cynomolgus monkeys with recombinant CD80 antigen, followed by cell fusion and cloning of the antibody-secreting heterohybridoma. The variable regions of the light and heavy chains were cloned and incorporated into a cassette vector containing human constant region genes. The primatized antibody, therefore, contains variable regions of cynomolgus macaque origin and constant regions of human origin. The N5LG1 vector, which encodes the antibody, is expressed in the Chinese hamster ovary transfectoma cell line DG44. The secreted antibody is subsequently purified from the medium using chromatography and filtration. Galiximab is formulated for human intravenous administration as a sterile product in a buffer containing sodium acetate 25 mmol/l, glycine 220 mmol/l, and 0.05% polysorbate 80 v/v at pH 6.0. CE9.1 (Biogen Idec), a primatized, anti-CD4 IgG1 mAb, served as an isotype-matched negative control. Fludarabine (NDC#0703-4852-11; Teva Parenteral Medicines Inc., Irvine, CA) and doxorubicin (NDC#55390-237-01; Bedford Labs, Bedford, OH) were the chemotherapeutic agents used.

### In vitro sensitization of Raji cells by galiximab to apoptosis by fludarabine or doxorubicin

Raji cells were treated with different concentrations of galiximab for 18 h and then treated with various concentrations of fludarabine or doxorubicin for an additional 18 h. The cells were harvested and examined by flow for apoptosis for the activation of caspase 3 as described previously (17).

### The human lymphoma subcutaneous tumor model

Mice with SCID were subcutaneously (s.c.) injected in the flank with Raji cells (2×10^6^) in 50% Matrigel basement membrane (BD Biosciences, Bedford, MA, USA) on day 0. After the tumors reached >100 mm^3^ in size, the mice were randomized into groups (n=10) and intraperitoneally injected with vehicle, control antibody (CE9.1), or various concentrations of galiximab (0.1, 1, 3 and 10 mg/kg) as a single agent to determine the optimum doses. Because the pharmacokinetic estimation indicated that galiximab has a half-life of 8.6 days (data not shown), galiximab was dosed once weekly. The mice received a total of 3 treatments. The tumors were measured biweekly with calipers and tumor volume was calculated using the formula: (length × width^2^)/2. The day-34 treatment effect was analyzed for statistical significance using an unpaired Student’s t-test with a 95% confidence interval.

In addition, to determine combination effects, when tumors reached 100–150 mm^3^ (early) or 200–400 mm^3^ (late), the mice were randomized into groups (n=10) and intraperitoneally injected with vehicle, control antibody (CE9.1), or galiximab (3 mg/kg per week). Some mice were then treated with intraperitoneal fludarabine (100 mg/kg per day) 3 days following the first antibody injection. These mice were treated with fludarabine for 5 consecutive days. Treatment effects were analyzed using an unpaired Student’s t-test with a 95% confidence interval.

### Disseminated human lymphoma model

SCID mice were intravenously injected in the tail vein with SKW6.4 lymphoma cells (4×10^6^ cells) on day 0. On day 3, the mice were randomized into groups (n=10) and intraperitoneally treated with vehicle, isotype-matched control antibody (CE9.1), or varying concentrations of galiximab (5, 10 mg/kg). A total of 4 injections was administered on days 3, 10, 17 and 24; the mice were evaluated daily for disease-free survival (DFS) and disease-related events. For the doxorubicin experiments, the tumors were implanted as mentioned above. On day 3, the mice were randomized into groups (n=10) and intraperitoneally injected with vehicle, control antibody (CE9.1), or galiximab (10 mg/kg per week). Re-treatment with antibody occurred on days 7, 10 and ≥24. Two days after the first antibody injection, a group of mice was intravenously injected with doxorubicin (2.5 mg/kg per week). Re-treatment with doxorubicin occurred on days 12 and 19. DFS and disease-related events were recorded using Kaplan-Meier survival analysis. Because the progression of disseminated disease subsequent to intravenous administration of malignant cells rapidly leads to death and is preceded by hind-limb paralysis, a disease-related event was defined as <20% weight loss (with muscle weakness) and neurologic motor deficit. The anti-tumor response was analyzed using a log-rank test with a 95% confidence interval, and data were analyzed using GraphPad Prism (GraphPad Software Inc., San Diego, CA).

## Results

### Galiximab demonstrates in vivo antitumor activity in a human lymphoma tumor xenograft model

**Figure 1.**
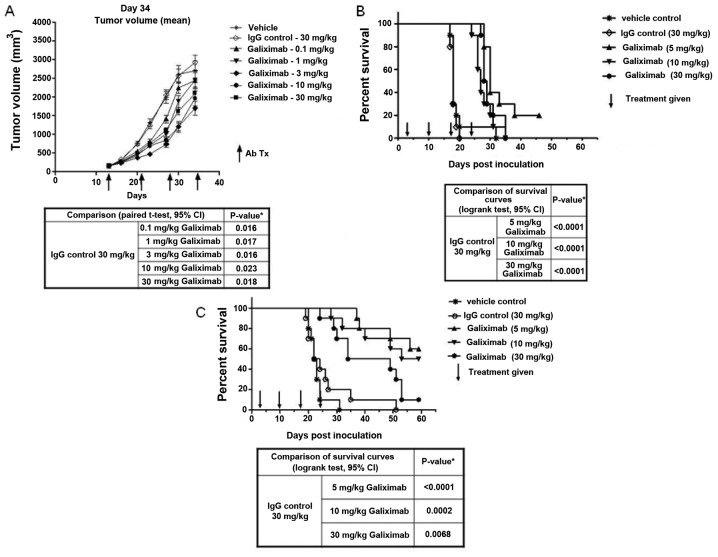
Galiximab demonstrates antitumor response and survival in solid tumor and disseminated lymphoma tumor models. (A and B) On day 0, SCID mice were inoculated subcutaneously in the flank with Raji lymphoma cells (2×10^6^) in 50% Matrigel. When the solid tumors reached 150 mm^3^, the mice were randomized into groups (n=10) and treated weekly with intraperitoneal injections of vehicle, control antibody or varying concentrations of galiximab (0.1–30 mg/kg) or isotype-matched control antibody (CE9.1) (30 mg/kg) for a total of 4 injections. Tumor volume was calculated twice weekly, from bidirectional caliper measurements using the formula: (length × width^2^)/2. On day 34, antitumor response was analyzed for statistical significance using an unpaired Student’s t-test with a 95% confidence interval (A). The mice were evaluated daily and disease events were recorded using Kaplan-Meier survival analysis (B). (C) On day 0, SKW lymphoma cells (4×10^6^) were intravenously inoculated into the tail vein of SCID mice. Three days after inoculation, the mice were randomized into groups (n=10) and intraperitoneally injected with vehicle, isotype-matched control (30 mg/kg), or varying concentrations of galiximab (5, 10 or 30 mg/kg) for a total of 4 weekly injections. The mice were evaluated daily, and disease events were recorded using Kaplan-Meier survival analysis. A disease event was categorized as <20% weight loss (with muscle weakness) and neurological motor deficit. The antitumor response was analyzed with a log-rank test with a 95% confidence interval.

The antitumor activity of galiximab was tested *in vivo* in mice bearing the Raji human tumor xenograft. SCID mice were s.c. injected *in vivo* in the flank with Raji cells (2×10^6^) in 50% Matrigel on day 0. After the tumor reached >100 mm^3^, the mice were randomized into groups (n=10) and injected intraperitoneally with vehicle, control IgG isotype (CE9.1), or various concentrations of galiximab (0.1, 1.0, 3.0, 10 and 30 mg/kg) as single agent. The mice received a total of three treatments with galiximab (d14, d21 and d28). The mice were measured bi-weekly with calipers and tumor volume was calculated as described in methods. The day 34 treatment effect was analyzed for statistical significance. The findings summarized in Fig. 1A demonstrate a dose-dependent decrease in the mean tumor volume. Tumor reduction reached statistical significance with all galiximab concentrations used (see table below Fig. 1A). Analysis of the survival of the treated mice revealed that there was a significant prolongation of survival in all mice treated with concentrations of galiximab ranging from 5–30 mg/kg (Fig. 1B, and table below).

The disseminated lymphoma tumor model SKW was examined. SCID mice were treated with various concentrations of galiximab (5, 10 and 30 mg/kg) and examined at 60 days for survival. There was significant prolongation of survival with all concentrations of galiximab used (Fig. 1C). The statistical analyses for significance are summarized in a table below Fig. 1C.

### Potentiation of tumor growth inhibition by the combination treatment of galiximab and fludarabine

**Figure 2.**
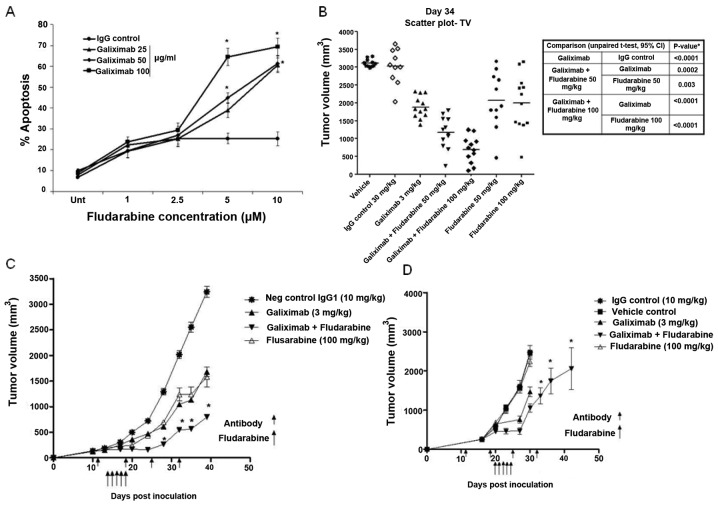
Galiximab sensitizes Raji cells to apoptosis by fludarabine and its antitumor effect *in vivo* by the combination treatment. (A) Raji cells were treated with various concentrations of galiximab (25, 50, 100 *μ*g/ml) or IgG control (100 *μ*g/ml) for 18 h and then treated with various concentrations of fludarabine (1–10 *μ*M) for an additional 18 h. The cells were then tested for apoptosis as described. (B) Mice were s.c. inoculated with Raji cells and treated with vehicle, IgG control (3 mg/kg), galiximab (3 mg/kg) and fludarabine (50 and 100 mg/kg). Tumor volumes were measured and recorded. (C and D) On day 0, SCID mice were subcutaneously inoculated in the flank with Raji cells (2×10^6^) in 50% Matrigel. When the solid tumors reached a size of 100–150 mm^3^ (C, early tumor) or 200–400 mm^3^ (D, advanced solid tumor), the mice were randomized into groups (n=10) and (1) intraperitoneally injected with control IgG 1 (10 mg/kg) or galiximab (3 mg/kg) per week for 4 weeks (2) fludarabine (100 mg/kg per day) beginning on the third day following the initial antibody injection for 5 consecutive days, injected five times (d14–19) and (3) combination of galiximab and fludarabine (3 mg/kg). The antitumor response was analyzed for statistical significance using an unpaired Student’s t-test with a 95% confidence interval.

We examined whether the combination treatment of galiximab and fludarabine may potentiate the antitumor response *in vivo. In vitro*, Raji cells were treated with various concentrations of galiximab (25, 50 and 100 *μ*g/ml) and with various concentrations of fludarabine (1–10 *μ*M) and the cells were analyzed for apoptosis as described in methods. The findings shown in Fig. 2A demonstrate that whereas treatment with either single agent alone resulted in modest apoptosis, however, the combination treatment resulted in significant potentiation of apoptosis.

These above *in vitro* findings prompted us to examine *in vivo* the effect of the combination treatment of galiximab and fludarabine on the growth of Raji tumor. Mice were treated with a single dose of galiximab (3 mg/kg) and two doses of fludarabine (50 and 100 mg/kg) and tumor volumes were measured. The findings in Fig. 2B demonstrate that whereas galiximab used alone or fludarabine used alone showed reduction in tumor volume, however, the combination treatment was more potent in reducing tumor weight. The statistical analyses are summarized in the table adjacent to Fig. 2B.

We then examined the efficacy of the above treatments in two different models with preexisting different tumor sizes. Two protocols were used, namely, treatment was initiated in mice with a tumor xenograft reaching either 100–180 or 200–400 mm^3^ prior to drug treatment for early or late advanced tumor models, respectively. In the early model, treatment with galiximab (3 mg/kg) or fludarabine (100 mg/kg) resulted in a significant decrease in tumor volume (p<0.001). However, treatment with the combination of galiximab and fludarabine improved the antitumor effect with a significant reduction of tumor volume when compared with treatment of galiximab alone (p<0.001) or fludarabine alone (p<0.001) as shown in Fig. 2C. In the late advanced tumor model, treatment with fludarabine alone had no effect. Treatment with galiximab retarded tumor growth. However, a specific tumor reduction was observed when galiximab was used in combination with fludarabine and the effect was statistically significant (p<0.001) (Fig. 2D).

### Tumor growth inhibition and survival in mice treated with galiximab and doxorubicin

**Figure 3.**
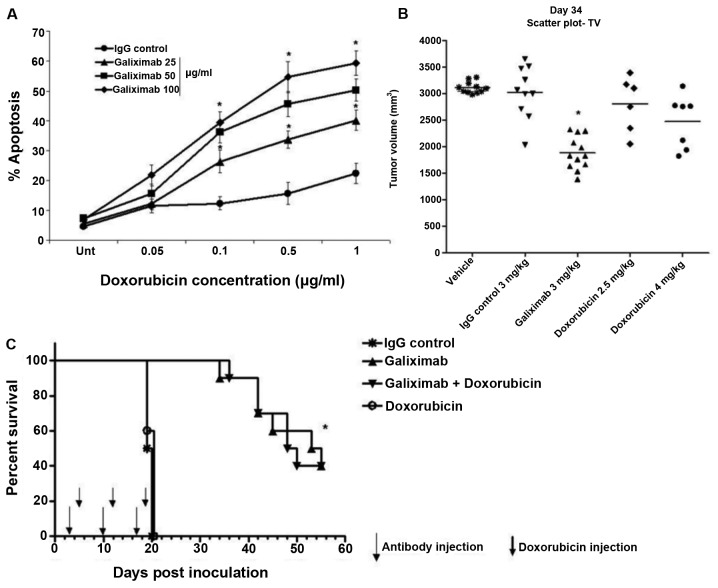
Galiximab sensitizes Raji cells to apoptosis by doxorubicin and its antitumor effect *in vivo* by the combination treatment. (A) Raji cells were treated with various concentrations of galiximab (25, 50 and 100 *μ*g/ml) or IgG control (100 *μ*g/ml) for 18 h and then treated with various concentrations of doxorubicin (0.05–1 *μ*g/ml) for an additional 18 h and apoptosis was measured as described in methods. (B) SCID mice bearing tumor xenografts were treated with vehicle, IgG control (3 mg/kg), galiximab (3 mg/kg) or doxorubicin (4 mg/kg). Tumor volumes were calculated as described in methods. (C) SCID mice bearing tumor xenografts were treated with galiximab, doxorubicin or combination, and Kaplan-Meier was used to analyze survival.

We first examined the cytotoxic effect observed following treatment of Raji cells with galiximab, doxorubicin, or combination. Raji cells were treated with various concentrations of galiximab (25, 50, 100 *μ*g/ml) for 18 h and treated for an additional 18 h with various concentrations of doxorubicin (0.05–1.0 *μ*g/ml) and apoptosis was determined as described in methods. While treatment with galiximab resulted in no effect, treatment with doxorubicin showed modest apoptosis (about 10%) at high concentrations. However, the combination treatment resulted in significant potentiation of apoptosis (Fig. 3A).

*In vivo*, the Raji tumor-bearing mice were treated with galiximab (3 mg/kg) or doxorubicin (2.5 and 4 mg/kg) and tumor growth was monitored. As expected, treatment with galiximab resulted in significant inhibition of tumor growth. However, there was modest antitumor effect by the treatment with doxorubicin alone (4 mg/kg) (Fig. 3B). Mice treated with the combination of galiximab and doxorubicin were also monitored for survival. Doxorubicin alone was not effective. However, galiximab alone or the combination of galiximab and doxorubicin revealed significant prologation of survival, though, doxorubicin did not augment the survival of the mice treated with galiximab alone (Fig. 3C).

## Discussion

Galiximab is a primatized mAb that is directed against CD80, an antigen that is expressed on the surface of many B cell lymphomas (9–12,18). Treatment of B cell tumors with galiximab induces cell death via ADCC (13). Recently, we have reported that galiximab triggers B-NHL cell lines and inhibits the constitutively activated survival/anti-apoptotic NF-κB pathway and downstream transcription factors that regulate drug resistance such as Snail and YYI. The inhibition of either Snail or YY1 expression and activity by siRNAs sensitized the tumor cells to CCDP and TRAIL-induced apoptosis (17). The present study examined *in vivo* in mice the antitumor activity of galiximab used alone or in combination with drugs such as fludarabine or doxorubicin. The findings clearly demonstrate that treatment with galiximab inhibited B-NHL tumor xenografts growth and prolonged survival and its antitumor activity was potentiated when combined with fludarabine. The antitumor activity *in vivo* by galiximab was shown both in a solid and disseminated B-NHL tumor xenografts.

*In vitro* findings demonstrated that treatment of B-NHL cells with galiximab inhibited cell proliferation (13). This *in vitro* finding was validated preclinically *in vivo* in SCID mice bearing B-NHL tumor xenografts. The extent of inhibition of tumor growth was the function of the galiximab dose used. Further, there was a significant prolongation of survival with galiximab treatment correlating with the inhibition of tumor growth. A comparison between the antitumor effect of galiximab alone and its administration post-tumor inoculation was examined in an early tumor model (100–180 mm^3^) and a late tumor model (>200 mm^3^) and the findings demonstrated significant inhibition of tumor growth in both models.

*In vitro* findings demonstrated that galiximab sensitized Raji cells to apoptosis by fludarabine. Accordingly, analysis *in vivo* was performed to validate the *in vitro* findings. Treatment with fludarabine had a modest antitumor activity, whereas, the combination of galiximab and fludarabine demonstrated a significant inhibition of tumor growth and which was more significant than tumor growth inhibition by galiximab monotherapy. The mechanism by which galiximab potentiates the activity of fludarabine *in vivo* may be due, in part, to the findings showing the galiximab signals B-NHL cells and inhibits the constitutively activated survival/anti-apoptotic NF-κB pathway and downstream inhibition of the resistant factors such as Bcl-2, BCL-xL, Snail and YY1 as recently reported by us (17).

The *in vitro* findings obtained with the sensitization to apoptosis by the combination of galiximab and fludarabine were confirmed with the combination of galiximab and doxorubicin. *In vitro* analysis revealed that treatment of Raji cells with galiximab sensitized the cells to apoptosis to doxorubicin. *In vivo* treatment with doxorubicin showed a modest inhibition of tumor growth; however, there was no significant survival of mice treated with doxorubicin. In contrast, whereas treatment by galiximab alone resulted in significant prolongation of survival, there was no potentiation by the combination with doxorubicin. These findings *in vivo* did not concur with the *in vitro* findings as expected. Further studies with different doses and schedules are necessary to show if additive or synergistic effects are achieved by the combination or galiximab and doxorubicin.

The *in vivo* findings observed in the immuno-compromised SCID mice ruled out a contribution by the host immune system. It is not clear whether a more significant antitumor response by galiximab alone or its combination with drugs might have been achieved. We have reported that treatment of B-NHL cells with rituximab sensitized the tumor cells to both FasL and TRAIL, ligands that are expressed on host effector cells such as T, NK and macrophages (19,20). Thus, it is likely that the antitumor response in immuno-competent mice might have been augmented by the additional contribution of host effector antitumor activity to the direct effect of the combination treatment with galiximab alone or with drugs.

Clinically, galiximab has been tested in a clinical trial of phase I and II studies in patients with indolent follicular lymphoma (FL). A dose escalation trial of galiximab treatment as single agent was found to be well tolerated, with modest antitumor activity in patients with relapsed FL (14). In another phase I/II trial of galiximab plus rituximab in a relapsed/refractory FL population demonstrated a 66% overall response rate (ORR); 33% partial response (PR) with a median PFS of 12.1 month (15). Based on these findings, the CALGB 50402, phase II trial of galiximab plus rituximab was undertaken in previously untreated FL patients (16). The findings showed that the administration of galiximab plus rituximab was well tolerated and appears efficacious in patients with low risk FLIPI scores. The clinical findings observed with the combination of galiximab and rituximab *in vivo* may be the result of several, although unclear, underlying mechanisms of action. Clearly, antibody-dependent cellular cytotoxicity (ADCC) and complement-dependent cellular cytotoxicity (CDC) play a role in the inhibition of cell proliferation and in direct cytotoxicity. In addition, we have reported that both galiximab (17) and rituximab (19–21) inhibit survival pathways in B-NHL cells leading to susceptibility of tumor cells to direct cytotoxicity by both chemotherapeutic drugs and by host immune effector cells bearing FasL or TRAIL. For CD80, the role of the tumor microenvironment cannot be ruled out since CD80 is expressed on the surface of tumor-associated macrophages (TAM) and the inhibition of CD80-CD28 interaction by galiximab interferes with TAM-mediated cytokine responses and proangiogenic signals, all of which induce antitumor activity (8).

The present findings suggest the potential clinical application of the combination of galiximab and fludarabine in the treatment of B-NHL. This treatment strategy may be a complement to the current rituximab/CHOP therapy. The present findings and those of others also suggest the potential use of the triple combination of galiximab, rituximab and chemotherapy in the treatment of patients who are poor responders.

## Abbreviations:

ADCC: antibody-dependent cell-mediated cytotoxicity;
B-NHL: B-non-Hodgkin’s lymphoma;
CDC: complement-dependent cytotoxicity;
CDDP: cis-diamminedichloroplatinum(II);
CTLA-4: cytotoxic T-lymphocyte antigen 4;
DLBCL: diffuse large B cell lymphoma;
TRAIL: tumor necrosis factor (TNF)-related apoptosis-inducing ligand;
siRNA: small interfering RNA;
FL: follicular lymphoma;
galiximab: anti-CD80 mAb;
mAb: monoclonal antibody;
SCID: severe combined immunodeficiency;
DFS: disease free survival;
NF-κB: nuclear factor κ-light-chain-enhancer of activated B cells;
rituximab: anti-CD20 mAb;
YY1: Yin Yang 1
